# *Anaplasma phagocytophilum* infection modulates expression of megakaryocyte cell cycle genes through phosphatidylinositol-3-kinase signaling

**DOI:** 10.1371/journal.pone.0182898

**Published:** 2017-08-10

**Authors:** Supreet Khanal, Hameeda Sultana, John D. Catravas, Jason A. Carlyon, Girish Neelakanta

**Affiliations:** 1 Department of Biological Sciences, Old Dominion University, Norfolk, VA, United States of America; 2 Center for Molecular Medicine, College of Sciences, Old Dominion University, Norfolk, VA, United States of America; 3 Frank Reidy Research Center for Bioelectrics, Old Dominion University, Norfolk, VA, United States of America; 4 School of Medical Diagnostic and Translational Sciences, College of Health Sciences, Old Dominion University, Norfolk, VA, United States of America; 5 Department of Microbiology and Immunology, Virginia Commonwealth University Medical Center, School of Medicine, Richmond, VA, United States of America; University of Minnesota, UNITED STATES

## Abstract

*Anaplasma phagocytophilum*, the agent of human granulocytic anaplasmosis infects neutrophils and other cells from hematopoietic origin. Using human megakaryocytic cell line, MEG-01, we show that expression of cell cycle genes in these cells are altered upon *A*. *phagocytophilum* infection. Expression of several cell cycle genes in MEG-01 cells was significantly up regulated at early and then down regulated at later stages of *A*. *phagocytophilum* infection. Lactate dehydrogenase (LDH) assays revealed reduced cellular cytotoxicity in MEG-01 cells upon *A*. *phagocytophilum* infection. The levels of both PI3KCA (p110 alpha, catalytic subunit) and PI3KR1 (p85, regulatory subunit) of Class I PI3 kinases and phosphorylated protein kinase B (Akt/PKB) and inhibitory kappa B (IκB) were elevated at both early and late stages of *A*. *phagocytophilum* infection. Inhibition of PI3 kinases with LY294002 treatment resulted in significant reduction in the expression of tested cell cycle genes, *A*. *phagocytophilum* burden and phosphorylated Akt levels in these MEG-01 cells. Collectively, these results suggest a role for PI3K-Akt-NF-κB signaling pathway in the modulation of megakaryocyte cell cycle genes upon *A*. *phagocytophilum* infection.

## Introduction

In the United States, human granulocytic anaplasmosis (HGA) is one of the most common tick-borne diseases [1, 2]. Previous studies have shown that up to 30% of human population in endemic areas may have been exposed to *A*. *phagocytophilum* infections [3, 4]. At least 15, 952 HGA cases have been reported since 1995 with a 12-fold increased rate in 2001–2011 [5]. Infections in many cases are asymptomatic [2, 3, 5, 6]. However, HGA infections could lead to severe illness and death in many individuals particularly that are immunocompromised [5]. The common clinical manifestations of HGA include fever, malaise, headache, and/or myalgia. However, arthralgia, nausea, vomiting or cough may occur in some severely infected persons. In addition, thrombocytopenia (reduced platelet numbers), leucopenia, anemia and/or elevated levels of liver enzymes are often evident in HGA cases [2, 5, 6].

In the mammalian hosts, *A*. *phagocytophilum* survives primarily in the neutrophils, where it enters membrane-bound vacuoles that do not fuse with lysosome, thereby protecting itself from host toxic components and degradation [7, 8]. In addition, *A*. *phagocytophilum* delays apoptosis of the neutrophils by modulation of multiple apoptotic pathways [9, 10]. Several studies have shown that *A*. *phagocytophilum* alters host gene expression for its survival and replication [10–15]. *A*. *phagocytophilum* is closely related to *Ehrlichia* species [6, 16, 17]. *Ehrlichia chaffeensis* is reported to alter cell cycle genes for its survival in human monocytic cell line [18]. *A*. *phagocytophilum* also infects and survives in other hematopoietic cells [5, 19, 20]. While much is known about the interactions of *A*. *phagocytophilum* with neutrophils, very little is known whether this bacterium alters cell cycle gene expression for its survival in hematopoietic cells.

Megakaryocytes are the precursor cells for the production of platelets [21]. Initially, megakaryocytes mature and differentiate in bone marrow [21]. Upon differentiation, megakaryocytes extend their cytoplasmic structures to form proplatelets that later form segments leading to the formation of platelets [21]. Due to difficulty in the isolation of homogenous populations of bone marrow megakaryoblast cells, the use of *in vitro* cell lines has greatly facilitated convenient experimental system for several studies [22]. Ogura et al., in 1985 [23] reported the first use of the megakaryoblastic leukemia cell line (MEG-01). The phenotypic properties of this cell line closely resemble megakaryocytes [23]. Several studies have used the leukemic megakaryoblastic cell line, MEG-01, to study differentiation and maturation of these cells to platelets or platelet-like particles [24–27]. In addition, MEG-01 cells have been used to study cell cycle regulation, particularly during endomitosis and polyploidy [28]. These studies provide a strong basis for the use of MEG-01 cells to study infection-associated changes in megakaryocytes. *A*. *phagocytophilum* uses sialylated ligands such as PSGL-1 to enter neutrophils [29]. A study from Granick et al., (2008) has reported that *A*. *phagocytophilum* strain NCH-1 readily infects MEG-01 by using PSGL-1 to enter these cells [30]. *A*. *phagocytophilum* infection failed to alter platelet formation, but was noted to decrease cell proliferation of MEG-01 cells [30]. The mechanism by which *A*. *phagocytophilum* infection decreases MEG-01 cell proliferation is currently not understood.

Studies on *A*. *platys*, the agent of infectious canine cyclic thrombocytopenia, have suggested that severity of thrombocytopenia is highest in the first infection cycle due to the direct injury to platelets by replicating organisms or due to immune-mediated mechanisms [31, 32]. In addition, other studies have indicated that *A*. *platys* may infect megakaryocytes in the bone marrow in addition to direct infection of platelets [33, 34]. The cause for thrombocytopenia in many of the HGA cases is not completely understood.

Studies using murine models such as SCID mice (that lack T and B cells) and splenectomized mice have suggested that immune-mediated destruction or splenic sequestration of cells, respectively, are unlikely events that could lead to thrombocytopenia [35, 36]. Moreover, it appears that thrombocytopenia begins sooner than detection of reduced platelet numbers in the periphery [35, 36]. Quantitative PCR analysis did not reveal any correlation between pathogen burden in the mice blood and thrombocytopenia [35, 37]. However, the bone marrow colony numbers strongly correlated with the thrombocytopenia in *A*. *phagocytophilum*-infected mice [35, 37]. In addition, studies have reported that *A*. *phagocytophilum* infection results in the production of myelosuppressive chemokines such as interleukin-8 homologs, macrophage inflammatory protein-2 and chemokine ligand 1 (CXCL1 or KC) in bone marrow [37, 38]. Collectively, these studies suggest that the temporal changes and shift in bone marrow cell populations but not the pathogen burden could contribute to infection-associated thrombocytopenia [35–40]. To further recognize the infection-associated changes that could lead to a shift in hematopoietic cell populations during thrombocytopenia, studies in understanding interactions of *A*. *phagocytophilum* with platelet precursor cells, such as megakaryocytic cells, are highly warranted. In this study, we show that *A*. *phagocytophilum* infection modulates expression of cell cycle genes differentially at early and late stages in MEG-01 cells through PI3 kinase signaling, presumably for its survival in these cells.

## Materials and methods

### Bacterial isolates and megakaryocytic cell line

*A*. *phagocytophilum* isolate HZ was used throughout the study and referred as *A*. *phagocytophilum*. Isolate HZ was a kind gift from Dr. Joao Pedra, University of Maryland School of Medicine, USA and was maintained as described [41]. HL-60 cells were obtained from Dr. Jason Carlyon, Virginia Commonwealth University Medical Center, USA and was maintained as described [42]. The human bone marrow derived megakaryoblast (MEG-01 cells) was purchased from American Type Culture Collection (ATCC) and maintained in RPMI 1640 (Invitrogen) medium containing 10% heat-inactivated fetal bovine serum (FBS, Sigma). MEG-01 cells were maintained at 37°C incubator with 5% CO_2_ supply.

### *In vitro* MEG-01 cell line infection

*A*. *phagocytophilum* HZ strain was maintained in human promyelocytic cell line (HL-60) and cell free bacteria isolated from these cells were used for *in vitro* infection studies as described [15, 42]. The percentage of HL60 cells infected with *A*. *phagocytophilum* was calculated by Giemsa staining procedure [43] using modified Giemsa stain (Sigma, USA). In addition, QRT-PCR analysis (example shown in Figure A in S1 File) was performed on both uninfected and *A*. *phagocytophilum*-infected HL60 cells before proceeding for infection of MEG-01 cells. *A*. *phagocytophilum*-infected HL-60 cells (60–70% infected) were centrifuged for 10 min at 4,000 rpm and cell pellets were re-suspended in 3 ml IMDM medium with 20% FBS, lysed by six passages through 25-guage, followed by six more passages through 27-guage needles. Lysed cells were centrifuged at 1,200 rpm for 3 min to obtain cell free bacteria in supernatants. The supernatant (200 μl/well) containing *A*. *phagocytophilum* was used for infection of MEG-01 cells. Both uninfected and *A*. *phagocytophilum*-infected cells were plated simultaneously at the same time for all experiments. At different time points (day 1, 3, 5, 7) post infection (p.i.) cells or culture supernatants were collected for further analysis. The cell culture media was not changed during the course of the experiment. For PI3 kinase-inhibitor studies, MEG-01 cells were pre-treated with 50 μM LY294002 (PI3 kinase inhibitor, EMD Biosciences, USA) for 4 hours, followed by infection with *A*. *phagocytophilum*. Infected cells were treated with similar amounts of DMSO as mock controls. The concentration of the LY294002 inhibitor used in this study had no or marginal effects on viability of cells at day 1 p.i, but had some cytotoxic effects at day 7 p.i. The MEG-01 infection experiments were performed three independent times with three replicates per each group. Representative microscopic pictures of the inhibitor-treated cells in comparison to mock-treated cells are shown in the Figure B in S1 File.

### LDH Cytotoxicity assay

The cytotoxicity assays for uninfected or *A*. *phagocytophilum*-infected MEG-01 cells were measured using lactate dehydrogenase (LDH) assay kit (Pierce, USA) and following manufacturer’s instructions. Briefly, on different days (1, 3, 5, 7) p.i., 50 μl of culture supernatants were transferred to a flat bottom 96 well plate (Corning, USA) and mixed with 50 μl of LDH kit reaction mixture. The plates were then incubated for 30 min at room temperature in dark. The reactions were later stopped by addition of 50 μl of stop solution. The absorbance of the reactions was then measured at 490 and 680 nm. Final absorbance values were calculated after subtracting background values from 490 and 680 nm. LDH experiments were performed three independent times with three replicates per each group. Similar procedure was adapted to measure LDH release from uninfected or *A*. *phagocytophilum*-infected cells (treated with LY294002 or mock control) collected at different time points (days 1, 3, 5, 7 p.i.).

### Trypan blue staining

Trypan blue (Trypan blue 0.4% solution, Gibco, USA) staining was performed to evaluate percentage of live cells in each experimental group. Briefly, 1 x 10^5^ MEG-01 cells were plated in triplicates and infected with *A*. *phagocytophilum* as described in the earlier section. Cells were washed with 1x phosphate buffer saline (PBS) and then Trypan blue solution (400 μl) was added to the wells and incubated for 10 min. The PBS along with trypan blue stain was removed and 200 μl of fresh 1x PBS was added to the wells. The wells were imaged with EVOS imaging system. Images (24 images from 8 fields/well) were captured and analyzed for number of live and dead cells. Total percentage of live cells was calculated using the following formula: (number of trypan blue negative cells/total number of cells) x 100.

### RNA and DNA extraction and quantitative real-time PCR (QRT-PCR) analysis

Total RNA from MEG-01 cells was extracted using the Aurum Total RNA mini kit (Bio-Rad, USA) following the manufacturer’s instructions. RNA was converted to cDNA using iSCRIPT cDNA synthesis kit (BioRAD, USA). The generated cDNA was used as a template for quantifying transcripts of all cell cycle genes. As an internal control and to normalize the amount of template, beta-actin was quantified. QRT-PCR was performed using CFX96 QPCR system (BioRad) and iQ-SYBR Green Supermix (BioRad, USA). To quantify *Anaplasma* burden, the genomic DNA from *A*. *phagocytophilum*–infected MEG-01 cells was extracted using DNeasy kit (QIAGEN) and processed for PCR with primers specific for the *A*. *phagocytophilum p44* gene. The levels of cell cycle genes mRNA or *A*. *phagocytophilum* burden was quantified relative to the actin levels in each sample. In the QRT-PCR reactions, standard curve was generated using 10-fold serial dilutions starting from 1 ng to 0.00001 ng of known quantities of respective fragments (Figure C in S1 File). Oligonucleotides used in this study are shown in the Table A in S1 File.

### Immunoblotting

Total lysates from uninfected or *A*. *phagocytophilum*-infected MEG-01 cells at different days post infection (p.i.) was prepared in modified-RIPA buffer (BioExpress) supplemented with EDTA-free protease and phosphatase inhibitor cocktail (Sigma, USA). Protein concentrations were determined by BCA protein assay kit (Pierce, USA). Thirty micrograms of total lysates from each group were loaded onto a 12% non-reducing SDS-PAGE gel and processed for immunoblotting. The PIK3CA, PIK3R1 and pIκB antibodies (Cell Signaling Technologies; USA) and the pAKT and the rabbit or mouse polyclonal anti-IgG HRP-conjugated antibodies (Santa Cruz Biotechnology Inc.; USA) were used. ECL reactions were performed using Advanced WesternBright ECL HRP substrate kit (Advansta, USA) and chemiluminescence reaction images were captured using Chemidoc MP imager (BioRad). The beta-actin levels in each sample were considered as loading controls. The Immunoblotting images shown in the figures are representative images from one set of complete experiment. Densitometry analysis was performed as described [44] using Image Lab 4.1 by measuring intensities of PI3 kinases or pAkt or pIκB bands relative to the actin band (loading control).

### Statistics

The statistical significance of differences observed in data sets was analyzed using GraphPad Prism6 software and Microsoft Excel 2016. For data to compare two means, the non-paired Student *t* test was performed. P values of <0.05 were considered significant in all tests. Error bars represents standard deviation from the mean. Wherever necessary, statistical test and P values are indicated.

## Results

### *A*. *phagocytophilum* infection reduces cell death in megakaryocytic cell line MEG-01

**Fig 1:**
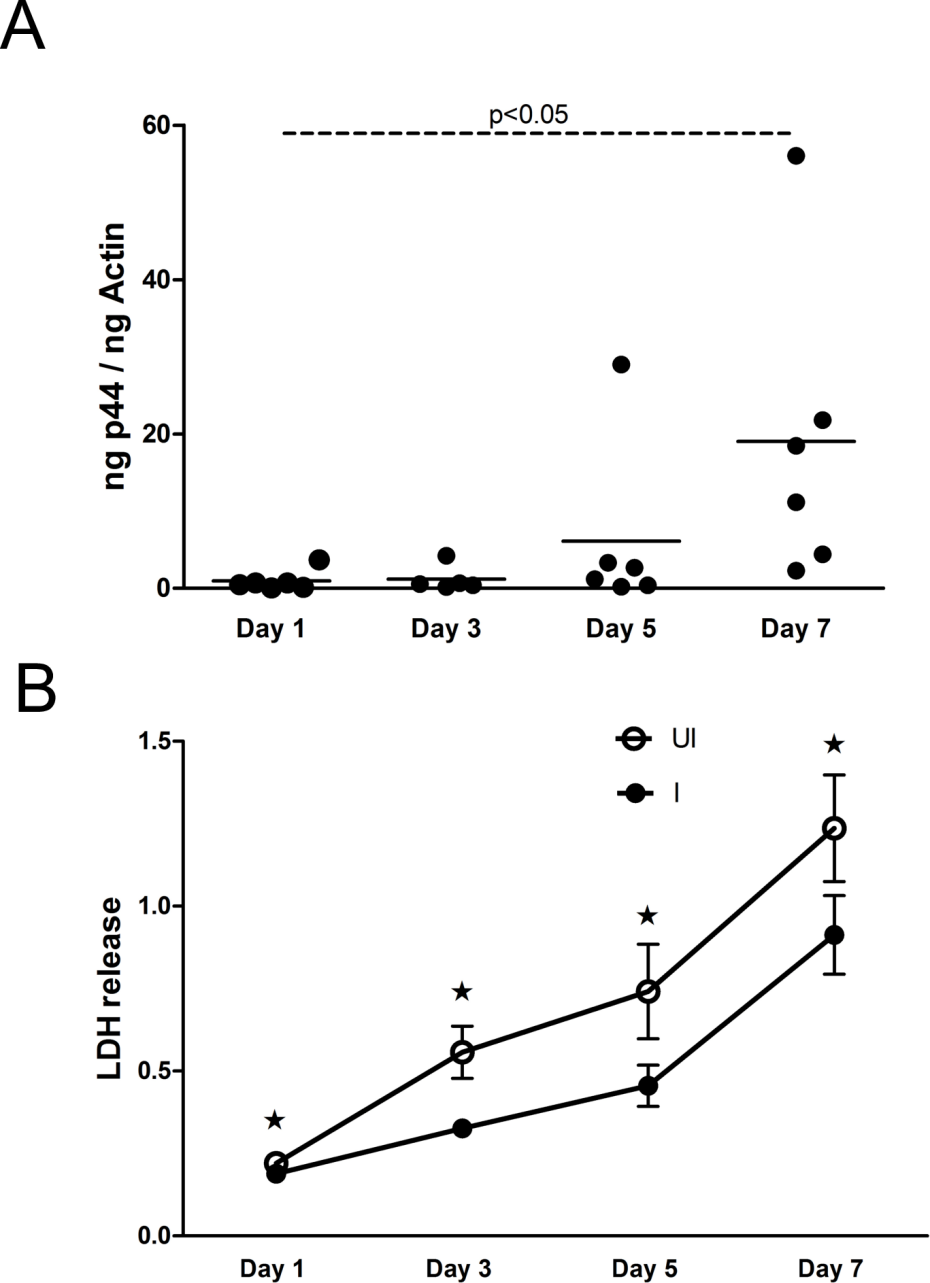
*A*. *phagocytophilum* infection decreases cytotoxicity in MEG-01 cells. **(**A) QRT-PCR analysis showing *A*. *phagocytophilum* burden in infected-MEG-01 cells at different days (1, 3, 5, 7) p.i. Each circle represents individual experimental sample. *A*. *phagocytophilum* specific *p44* DNA levels were normalized to human beta-actin levels. (B) LDH assay results performed with cell culture supernatants collected from uninfected (UI) or *A*. *phagocytophilum*-infected (I) at different days (1, 3, 5, 7) p.i. is shown. Values on the Y-axis show the absorbance for LDH release in the cell culture supernatants. The results for day 1 and day 7 are from three independent experiments and day 2 and 5 are from two independent experiments performed in triplicates. All cultures were started simultaneously at the same time. The culture medium was not changed daily. The P value (P<0.05) calculated from Student’s t test was considered significant. Asterisk above the error bar indicates statistically significant difference between UI and I samples from the same time point.

Previous study has shown that *A*. *phagocytophilum* strain NCH-1 readily infects MEG-01 cells [30]. We found similar infection kinetics with *A*. *phagocytophilum* HZ strain in MEG-01 cells (Fig 1A). QRT-PCR analysis revealed that *A*. *phagocytophilum* burden was significantly (P<0.05) higher at day 7 p.i. in comparison to day 1 p.i. of MEG- 01 cells (Fig 1A). However, no significant differences in the *A*. *phagocytophilum* burden were observed between days 1 p.i. and days 3 or 5 p.i. Due to the significant difference in the bacterial burden observed between two time points, the day 1 p.i. time point was considered as an early stage and day 7 p.i. as a late stage of *A*. *phagocytophilum* infection of MEG-01 cells.

We then tested whether *A*. *phagocytophilum* infection has any cytotoxic effects in megakaryocytes. The MEG-01 cell culture media was not changed daily during experiments and supernatants were collected from *A*. *phagocytophilum*-infected or uninfected MEG-01 cells at various time points p.i. and analyzed for lactate dehydrogenase activity (LDH cytotoxic assay). The duration of cell-media exposure for each time point corresponds to the respective day of p.i. Increased release of LDH is an indication of higher cytotoxicity. The assays performed with supernatants collected from *A*. *phagocytophilum*-infected MEG-01 cells at various time points (days 1, 3, 5, 7 p.i.) showed significantly (P<0.05) reduced LDH release from these cells at all tested time points in comparison to the respective uninfected controls (Fig 1B). In addition, trypan-blue staining also revealed significantly (P<0.05) increased number of live *A*. *phagocytophilum*-infected MEG-01 cells at days 5 and 7 p.i. time points in comparison to uninfected controls (Figure D in S1 File).

### *A*. *phagocytophilum* infection modulates expression of cell cycle genes in megakaryocytic cell line MEG-01

**Fig 2:**
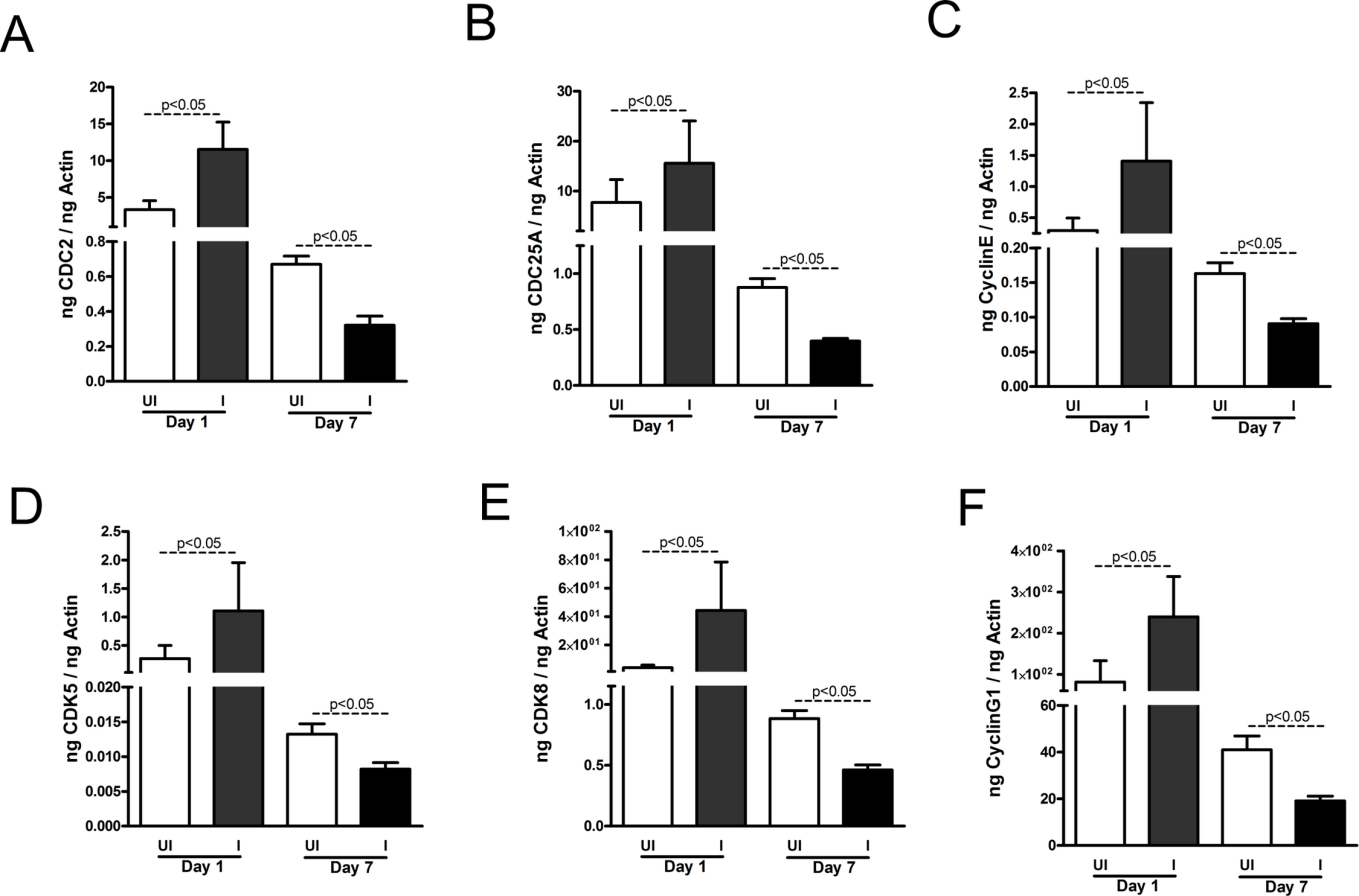
*A*. *phagocytophilum* infection alters cell cycle gene expression in MEG-01 cells. QRT-PCR analysis showing levels of CDC2 (A), CD25A (B), cyclin E (C), CDK5 (D), CDK8 (E) and cyclin G1 (F) in uninfected (UI) and *A*. *phagocytophilum*-infected (I) MEG-01 cells at days 1 and 7 p.i. The mRNA levels of cell cycle genes were normalized to human beta-actin mRNA levels. QRT-PCR was performed on samples generated from three independent experiments (3 samples/experiment) and analyzed in duplicates. The P value (P<0.05) calculated from Student’s t test was considered significant.

We then tested whether reduced cellular toxicity in MEG-01 cells is due to *A*. *phagocytophilum* infection-associated changes in the cell cycle regulation. QRT-PCR analysis revealed that at early stage of infection (day 1 p.i.), mRNA levels of cell cycle genes such as CDC2 (Fig 2A), CDC25A (Fig 2B), Cyclin E (Fig 2C), CDK5 (Fig 2D), CDK8 (Fig 2E) and Cyclin G1 (Fig 2F) were significantly (P<0.05) upregulated in *A*. *phagocytophilum*-infected cells in comparison to uninfected controls. However, at late stage of infection (day 7 p.i.), mRNA levels for all tested cell cycle genes were significantly (P<0.05) down regulated in *A*. *phagocytophilum*-infected cells in comparison to the uninfected MEG-01 cells (Fig 2A–2F). The evaluation of beta-actin transcripts normalized to total RNA concentrations revealed no significant (P>0.05) difference between uninfected and *A*. *phagocytophilum*-infected MEG-01 cells at both tested time (days 1, 7 p.i.) points (Figure E in S1 File). In addition, no significant (P>0.05) difference was evident in neither uninfected nor in *A*. *phagocytophilum*-infected MEG-01 cells between day 1 and day 7 p.i. time points (Figure E in S1 File). The pattern of variable expression of cell cycle genes in MEG-01 cells at both stages of *A*. *phagocytophilum* infection was not observed with housekeeping genes such as GAPDH (Figure E in S1 File), beta-tubulin (Figure E in S1 File) and glucose-6-isomerase (Figure E in S1 File). Collectively, these results indicate that megakaryocytic cell cycle gene expression is specifically and variably altered at different stages of *A*. *phagocytophilum* infection.

### *A*. *phagocytophilum* infection modulates expression of class I PI3 kinases in megakaryocytic cell line MEG-01

**Fig 3:**
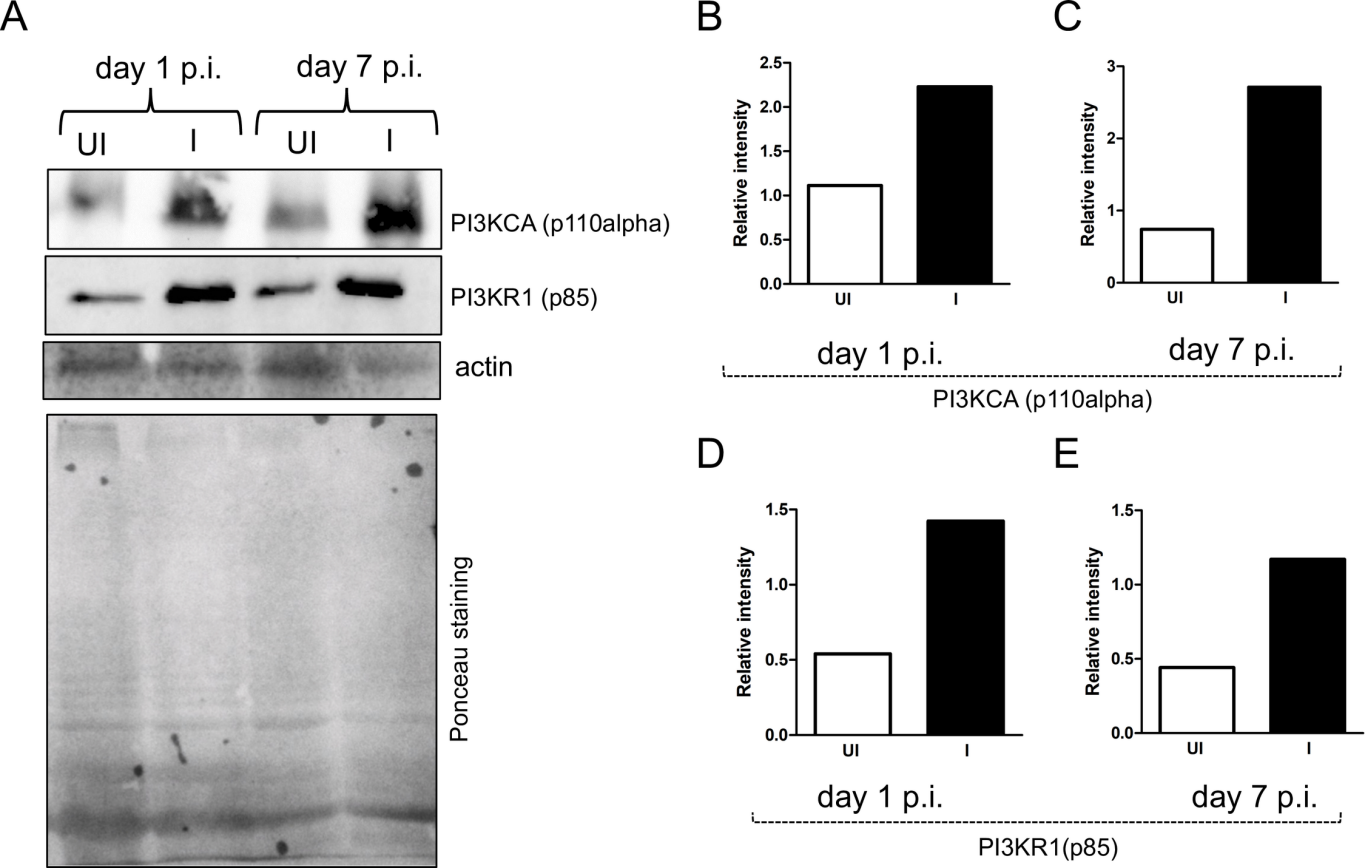
Altered expression of PI3KCA and PI3KR1 upon *A*. *phagocytophilum* infection of MEG-01 cells. **(**A) Immunoblotting analysis showing levels of PI3KCA, PI3KR1 in uninfected (UI) or *A*. *phagocytophilum*-infected (I) MEG-01 cells at days 1 and 7 p.i. The levels of actin (detected by immunoblotting) and Ponceau stained gel image for total protein serves as loading control in the immunoblotting analysis. (B) Densitometry analysis showing levels of PI3KCA (B and C) or PI3KR1 (D and E) at days 1 (B and D) and 7 (C and E) p.i. relative to the respective actin bands seen in A.

Studies have shown that *A*. *phagocytophilum* infection enhances PI3 kinase levels in human neutrophils [14, 45]. We therefore tested whether *A*. *phagocytophilum* infection affects expression of PI3KCA (p110 alpha catalytic subunit) and PI3KR1 (p85 regulatory subunit) of Class I PI3 kinases in MEG-01 cells. Immunoblotting assays revealed increased (~ 2 fold, as measured by densitometry analysis) levels of PI3KCA (Fig 3A, 3B and 3C) and PI3KR1 (Fig 3A, 3D and 3E) at both time points in *A*. *phagocytophilum*-infected MEG-01 cells in comparison to uninfected controls. These results suggest a role for Class I PI3 kinases at both early and late stages of *A*. *phagocytophilum* infection of megakaryocytic cells.

### Inhibition of PI3 kinases affects bacterial burden and infection-associated changes in cell cycle gene expression in megakaryocytic cell line MEG-01

**Fig 4:**
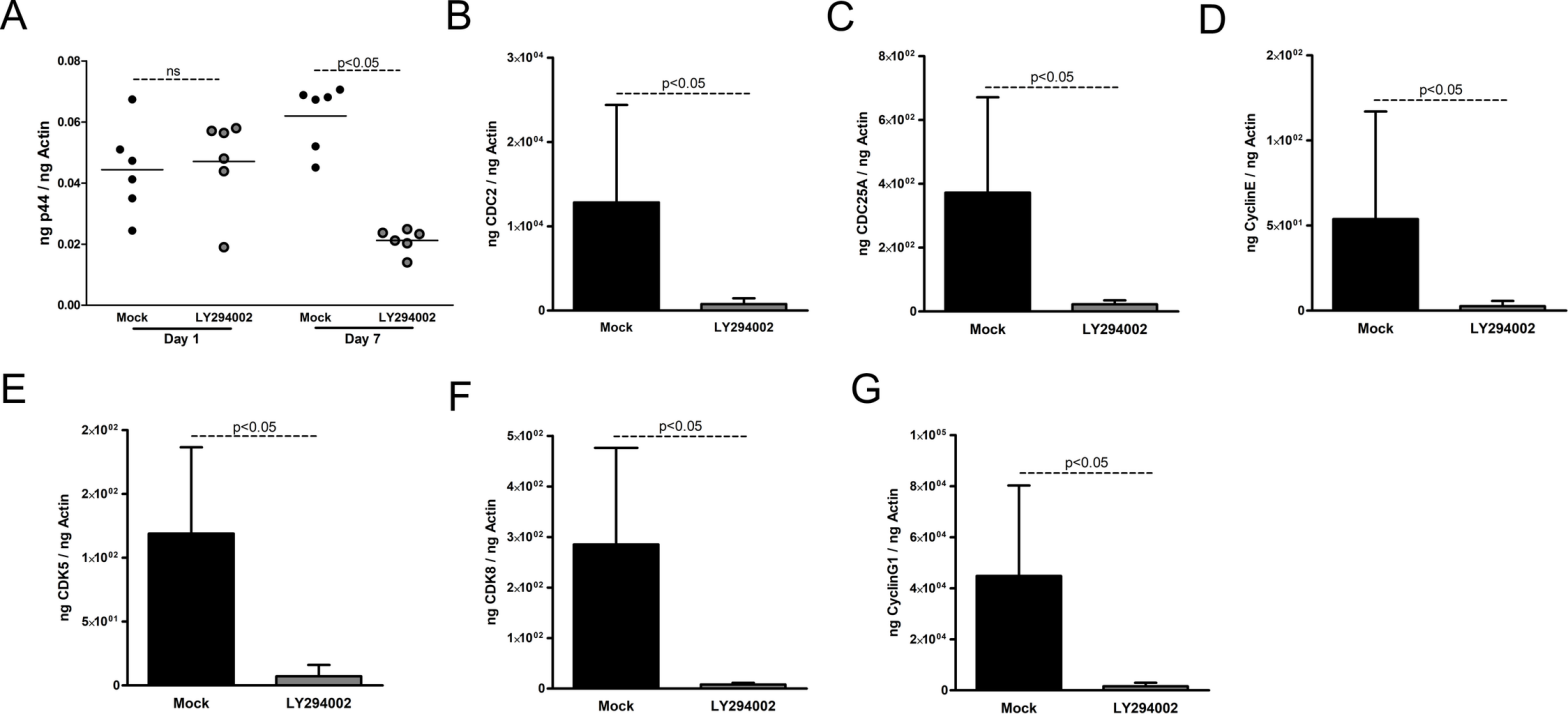
Inhibition of PI3 kinases reduces expression of cell cycle genes and *A*. *phagocytophilum* burden in MEG-01 cells. **(**A) QRT-PCR analysis showing bacterial burden in *A*. *phagocytophilum*-infected mock (DMSO-treated) or LY294002 (PI3K inhibitor)-treated MEG-01 cells at days 1 and 7 p.i. The *A*. *phagocytophilum* specific p44 DNA loads were normalized to human beta-actin levels. Each circle represents one individual sample. QRT-PCR analysis showing levels of CDC2 (B), CD25A (C), cyclin E (D), CDK5 (E), CDK8 (F) and cyclin G1 (G) in *A*. *phagocytophilum*-infected mock or LY294002 (PI3K inhibitor)-treated MEG-01 cells at day 7 p.i. QRT-PCR was performed on samples generated from three independent experiments (3 samples/experiment) and analyzed in duplicates. The levels of cell cycle genes were normalized to human beta-actin. The P value (P<0.05) calculated from Student’s t test was considered significant.

To test whether *A*. *phagocytophilum* infection modulates cell cycle gene expression in megakaryocytic cell line through PI3 kinase signaling, we treated *A*. *phagocytophilum*-infected MEG-01 cells with LY294002, a morpholine-containing chemical compound and a strong inhibitor of PI3 kinases including Class I PI3 kinases. *A*. *phagocytophilum*-infected MEG-01 cells treated with mock solution (DMSO) were used as controls. No significant (P>0.05) difference in *A*. *phagocytophilum* burden was evident at day 1 p.i. in LY294002-treated MEG-01 cells in comparison to the mock-treated control (Fig 4A). However, *A*. *phagocytophilum* burden was significantly reduced at day 7 p.i. in LY294002-treated MEG-01 cells in comparison to the mock-treated control (Fig 4A). In addition, significant (P<0.05) reduction in the expression of CDC2 (Fig 4B), CDC25A (Fig 4C), Cyclin E (Fig 4D), CDK5 (Fig 4E), CDK8 (Fig 4F) and Cyclin G1 (Fig 4G) was noted in *A*. *phagocytophilum*-infected LY294002-treated cells at day 7 p.i in comparison to the mock-treated control. These results suggest a role for class I PI3 kinases in *A*. *phagocytophilum* infection-associated changes in the cell cycle gene regulation in MEG-01 cells.

### Inhibition of PI3 kinases affects infection-associated reduced cytotoxicity in megakaryocytic cell line MEG-01

**Fig 5:**
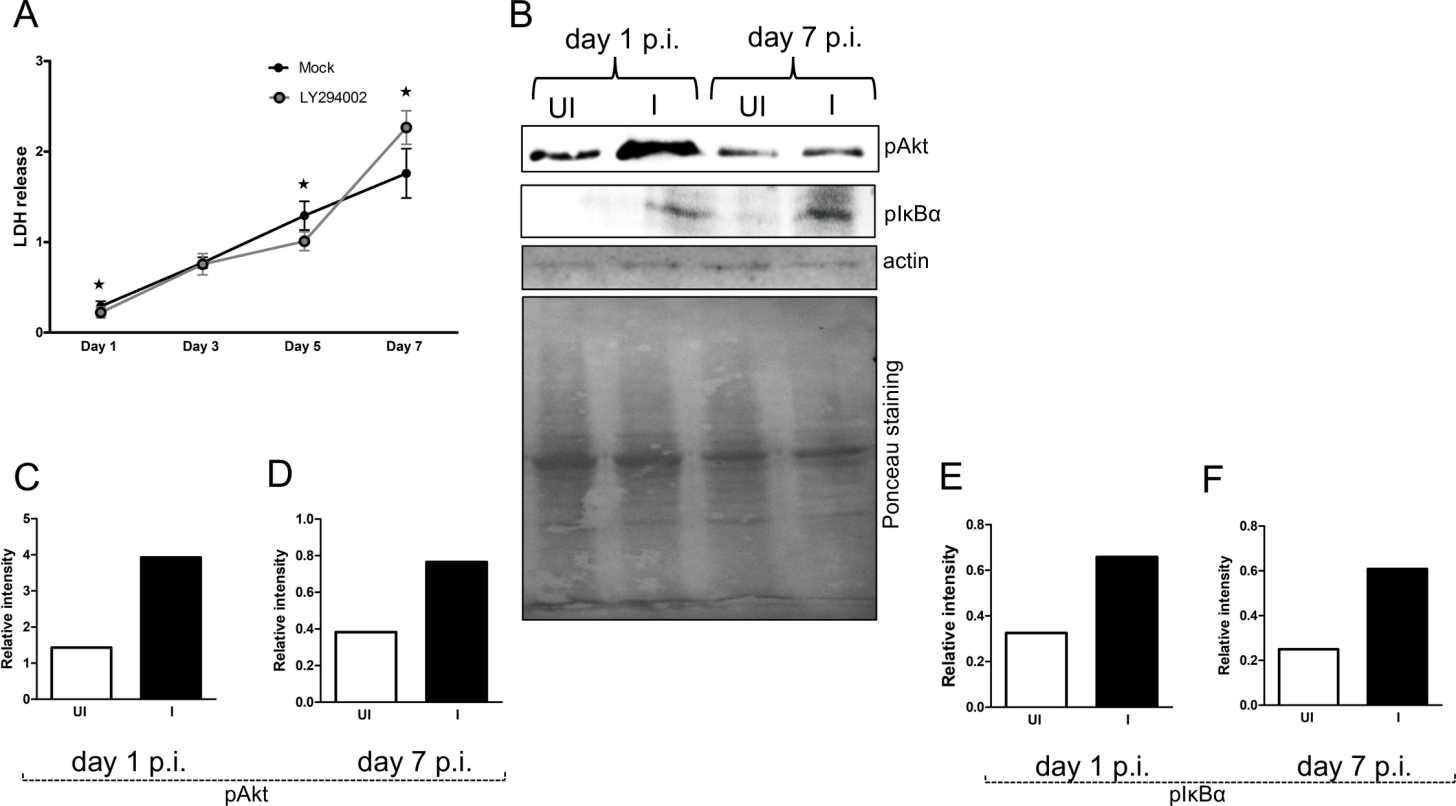
*A*. *phagocytophilum* infection increases Akt and NF-κB activation in MEG-01 cells. **(**A) LDH assay results performed with cell culture supernatants collected from *A*. *phagocytophilum*-infected mock-treated (mock) or *A*. *phagocytophilum*-infected LY294002 (PI3K inhibitor)-treated at different days (1, 3, 5, 7) p.i is shown. Values on the Y-axis show the absorbance for LDH release in the cell culture supernatants. The results are from three independent experiments performed in triplicates. All cultures were started simultaneously at the same time. The culture medium was not changed daily. The P value (P<0.05) calculated from Student’s t test was considered significant. (B) Immunoblotting analysis showing levels of phosphorylated Akt and IκB alpha in uninfected (UI) or *A*. *phagocytophilum*-infected (I) MEG-01 cells at days 1 and 7 p.i. The levels of actin (detected by immunoblotting) and Ponceau stained gel image for total protein serves as loading control. Densitometry analysis showing levels of Akt (C and D) or IκB alpha (E and F) at days 1 (C and E) and 7 (D and F) p.i., relative to the respective actin bands seen in B.

We then analyzed whether PI3 kinases are involved in the increased cell survival of MEG-01 cells upon *A*. *phagocytophilum* infection. LDH assays performed with supernatants collected from *A*. *phagocytophilum*-infected MEG-01 cells treated with LY294002 at days 1, 3, 5 p.i. showed significantly (P<0.05) reduced LDH release in comparison to the mock-treated cells (Fig 5A). However, assays performed with supernatants collected from *A*. *phagocytophilum*-infected MEG-01 cells treated with LY294002 at late time point (day 7 p.i.) showed increased (P<0.05) LDH release in comparison to the mock-treated cells (Fig 5A). These results indicate that the reduction in the cellular toxicity in MEG-01 cells at later stages of *A*. *phagocytophilum* infection is mediated through class I PI3 kinase signaling.

### *A*. *phagocytophilum* infection modulates PI3K-Akt-NF-κB signaling in megakaryocytic cell line MEG-01

**Fig 6:**
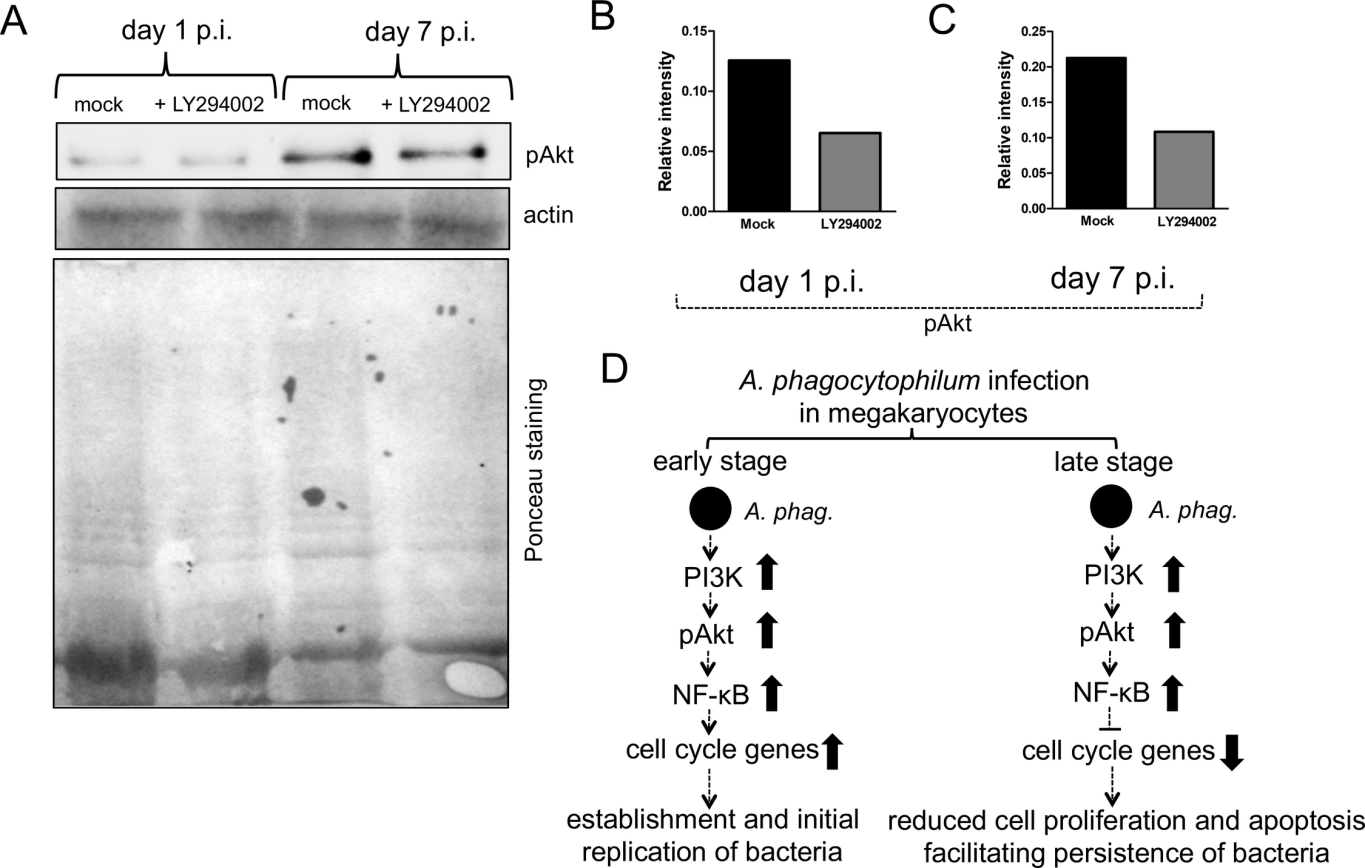
*A*. *phagocytophilum* infection modulates PI3K-Akt-NF-κB signaling in MEG-01 cells. (A) Immunoblots showing levels of phosphorylated Akt in *A*. *phagocytophilum*-infected mock or LY294002-treated MEG-01 cells at days 1 and 7 p.i. The levels of actin (detected by immunoblotting) and Ponceau stained gel image for total protein serves as loading control. Densitometry analysis showing levels of phosphorylated Akt (B and C) at days 1 (B) and 7 (C) p.i., relative to the respective actin bands seen in A. (D) Model suggesting impact of *A*. *phagocytophilum*-infection on the expression of megakaryocyte cell cycle genes at early and late stages of infection. Upon *A*. *phagocytophilum* infection, increased PI3K activity levels lead to enhanced Akt activation that subsequently increases NF-κB activation. The activated NF-κB translocates to nucleus and may directly or indirectly upregulate cell cycle gene expression at early stage of infection or downregulate in the later stages of *A*. *phagocytophilum* infection. The upregulation of cell cycle genes at early stage of infection could facilitate *A*. *phagocytophilum* to initially establish and replicate. The downregulation of cell cycle genes at later stage of infection could protect cells from apoptosis and allow bacterium to persist for a longer period of time in these cells.

*A*. *phagocytophilum* activates PI3K/Akt and NF-κB survival pathways in neutrophils [46]. NF-κB is an important transcriptional regulator of cell cycle genes [47]. We therefore tested whether upregulation of PI3KCA and PI3KR1 upon *A*. *phagocytophilum* infection of MEG-01 cells (Fig 3) activates Akt that subsequently lead to NF-κB activation. Immunoblotting results revealed that upon *A*. *phagocytophilum* infection of MEG-01 cells, approximately 2-3-fold increased level of phosphorylated Akt (Fig 5B–5D) and IκB (Fig 5B, 5E and 5F) was evident at both day 1 (Fig 5B, 5C and 5E) and day 7 (Fig 5B, 5D and 5F) p.i. time points. However, upon inhibition of PI3K kinases, reduced (~ 2-fold) level of phosphorylated Akt was observed in *A*. *phagocytophilum*-infected LY294002-treated cells in comparison to mock control at both time points (Fig 6A–6C). Collectively, these results suggest that differential modulation of cell cycle gene expression and reduction in cellular toxicity in megakaryocytes upon *A*. *phagocytophilum* infection is mediated through PI3K-Akt-NF-κB signaling.

## Discussion

Human pathogens have developed variety of strategies to manipulate host-cell functions, including influence on cell cycle, presumably for their own benefit. The observation of differential modulation of cell cycle gene expression upon *A*. *phagocytophilum* infection of megakaryocytic MEG-01 cells suggests an interesting model to understand host-pathogen interactions in these cells of hematopoietic origin.

Several studies have shown that PI3 kinases play multiple roles in cell cycle regulation and cell survival [48–50]. The increased levels of Class I PI3 kinase subunits PIK3CA and PI3KR1 suggest increased PI3 kinase activity at both early and late stages of *A*. *phagocytophilum* infection of MEG-01 cells (Fig 6D). Increased phosphorylated Akt levels (Fig 5B–5D), an indication of increased activated Akt, in *A*. *phagocytophilum*-infected MEG-01 cells further support this notion. Moreover, reduced phosphorylated Akt levels in LY294002-treated cells further support the role for Class I PI3 kinases in the activation of Akt upon *A*. *phagocytophilum* infection of MEG-01 cells. PI3K/Akt pathway regulates NF-κB activity [51]. NF-κB is a transcription factor that regulates variety of genes involved in cell cycle, apoptosis, proliferation, differentiation and host immune responses [47, 52]. In the non-activated form, NF-κB is found to be associated with inhibitor IκB [53]. However, during activation of NF- κB, the IκB gets phosphorylated for polyubiquitination and degradation. This modification of IκB allows free NF-κB to translocate to nucleus for transcription. The observation of increased IκB levels suggests that PI3K/Akt activation subsequently influence NF-kB activation to differentially regulate cell cycle genes upon *A*. *phagocytophilum* infection of MEG-01 cells (Fig 6D).

The role of NF-κB in the induction (at early stage) and repression (at late stage) of expression of cell cycle genes upon *A*. *phagocytophilum* infection of MEG-01 cells is not surprising. In fact, NF-κB activation has been linked to inducing the expression of anti-apoptotic and apoptotic genes for both cell survival and death [47, 54]. NF-κB activation is shown to progress cell cycle through cyclin D_1_, a cyclin crucial for DNA synthesis [47] and arrest cell cycle in G2-M phase through p21^waf1/cip1^ induction [54]. Megakaryocytes undergo endomitosis, where these cells mature to a polyploidy state as a result of DNA replication in the absence of mitosis [55]. Various studies have characterized the roles for cyclin D and E (G1/S-phase regulators) in a view to support the hypothesis that up-regulation of these components are critical for promoting endomitosis to allow megakaryocytes to achieve high polyploidy state [56–58]. As megakaryocytes are committed to differentiate rather than to remain in G0 phase, the enodomitosis and polyploidization are very important for the cytoplasmic maturation [55]. The theoretical relationship between high nuclear (DNA and RNA) and protein content in polyploidy megakaryocytes and efficient platelet formation is still unclear. Our study suggests that NF-κB-dependent induction of cell cycle gene expression at early stages of *A*. *phagocytophilum* infection could lead to increased progression of MEG-01 cells to polyploidy state that may facilitate initial replication and establishment of bacteria in these cells (Fig 6D). In addition, the increased polyploidy state of megakaryocytes could lead to high accumulation of DNA facilitating elevated mRNA or protein content of host genes required for bacterial establishment. At late stages of *A*. *phagocytophilum* infection, NF-κB-dependent repression of cell cycle gene expression could lead to cell cycle arrest and protect cells from apoptosis, thereby facilitating bacterial survival in these cells (Fig 6D). The findings from the current study that indicate reduced cellular toxicity in MEG-01 cells at later time points and observation of reduced proliferation of these cells upon *A*. *phagocytophilum* infection [30] further supports this hypothesis. The NF-κB dependent cell cycle arrest upon *A*. *phagocytophilum* infection may lead megakaryocytes in a stationary stage that could facilitate persistence of this bacterium for a longer period of time in these cells before escaping to infect platelets or other cells of hematopoietic origin. Overall, this study provides an interesting model to understand the modulation of cell cycle genes in the persistence of a human pathogen in megakaryocytes.

## Conclusions

In this study, we show that upon *A*. *phagocytophilum* infection, the megakaryocyte cell cycle gene expression is altered via PI3K-Akt-NF-κB pathway and inhibition of PI3 kinases affects bacterial burden in these cells. This work not only increases our knowledge of *A*. *phagocytophilum* infection but may also lead to the development of new strategies to interrupt the survival of this bacterium in the cells of hematopoietic origin.

## Supporting information

S1 File Figure A. *A*. *phagocytophilum* loads in HL-60 cells. Figure B. Inhibition of PI3 kinases by LY294002 treatment at 50 μM concentration had no or some cytotoxicity on viability of MEG-01 cells. Figure C. Amplification of cell cycle genes by polymerase chain reaction (PCR). Figure D. Viability of MEG-01 cells upon *A*. *phagocytophilum* infection. Figure E. Expression of housekeeping genes upon *A*. *phagocytophilum* infection of MEG-01 cells. Table A. Primer sequences used in QRT-PCR analysis.
(PDF)

## Abbreviations

MEG-01: Megakaryoblast cell line
PI3K: phosphatidylinositol-3-kinase
PI3KCA: PI3 kinase p110 alpha catalytic subunit
PI3KR1: PI3 kinase p85 regulatory subunit
Akt: serine/threonine kinase or protein kinase B
pAkt: phospho Akt
NF-kB: nuclear factor kappa-light-chain-enhancer of activated B cells
PCR: Polymerase chain reaction
QRT-PCR: Quantitative real-time polymerase chain reaction
LDH: lactate dehydrogenase
p.i.: post infection
h: hours
ns: not significant
