# Supplementary material for: Anaplasma phagocytophilum infection modulates expression of megakaryocyte cell cycle genes through phosphatidylinositol-3-kinase signaling
Source: PLoS One. 2017 Aug 10;12(8):e0182898. doi: 10.1371/journal.pone.0182898 (PMC5552339; doi:10.1371/journal.pone.0182898)
Supplement: S1 File — Figure A. A. phagocytophilum loads in HL-60 cells. Figure B. Inhibition of PI3 kinases by LY294002 treatment at 50 μM concentration had no or some cytotoxicity on viability of MEG-01 cells. Figure C. Amplification of cell cycle genes by polymerase chain reaction (PCR). Figure D. Viability of MEG-01 cells upon A. phagocytophilum infection. Figure E. Expression of housekeeping genes upon A. phagocytophilum infection of MEG-01 cells. Table A. Primer sequences used in QRT-PCR analysis. (PDF) [file pone.0182898.s001.pdf]

Anaplasma phagocytophilum **infection modulates expression of megakaryocyte cell cycle genes through phosphatidylinositol-3-kinase signaling**

Supreet Khanal [1], Hameeda Sultana [1, 2], John D. Catravas [3, 4], Jason A. Carlyon [5] and Girish Neelakanta [1, 2, #]

Supplementary information

Supplementary Figure A. *A. phagocytophilum* loads in HL-60 cells. QRT-PCR analysis showing *A. phagocytophilum* burden in infected-HL-60 cells before isolation of bacteria. *A. phagocytophilum* specific *p44* DNA levels were normalized to total DNA levels. Uninfected cells were used as negative controls.

Supplementary Figure B. Inhibition of PI3 kinases by LY294002 treatment at 50 µM concentration had no or some cytotoxicity on viability of MEG-01 cells. Phase contrast microscopic images of *A. phagocytophilum*-infected mock-treated (DMSO) or LY294002-treated (PI3K inhibitor; at 50 µM concentration) MEG-01 cells, captured using EVOS FL cell imaging system (Invitrogen, USA) on different days (1 and 7 p.i.) are shown. Two images from each group and at each time point are shown. Scale bar represents 200 µm.

Supplementary Figure C. Amplification of cell cycle genes by polymerase chain reaction (PCR). Agarose gel images showing products from PCR amplification of cell

cycle genes or human beta actin for the generation of QRT-PCR standards. Total RNA generated from uninfected (UI) MEG-01 cells was converted to cDNA and used as template for generation of these fragments. Primers specific for each gene are shown in Supplementary table 1. NTC indicates no template control.

Supplementary Figure D. Viability of MEG-01 cells upon *A. phagocytophilum* infection. Trypan blue staining results performed with uninfected or *A. phagocytophilum*-infected MEG-01 cells at different days (1, 3, 5, 7) p.i is shown. Values on the Y-axis show the percentage of live cells. The percentage data is the average reading of 24 microscopic fields obtained from 3 wells per group. The P value ($P<0.05$) calculated from Student's t test was considered significant.

Supplementary Figure E. Expression of housekeeping genes upon *A. phagocytophilum* infection of MEG-01 cells. QRT-PCR analysis showing levels of actin, GAPDH, beta-tubulin and glucose-6-isomerase in uninfected (UI) and *A. phagocytophilum*-infected (I) MEG-01 cells at days 1 and 7 p.i. The mRNA levels of human beta-actin were normalized to total RNA levels. The mRNA levels of other three genes were normalized to human beta-actin mRNA levels. ns indicates no significance ($P>0.05$) calculated from Student's t test.

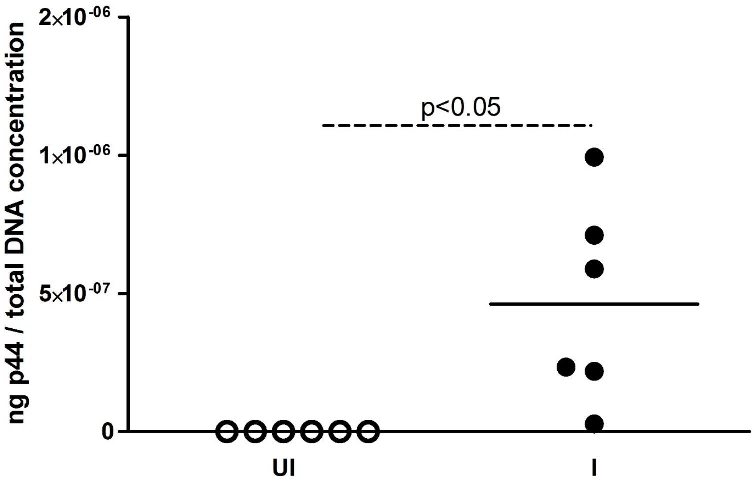

Supplementary Figure A

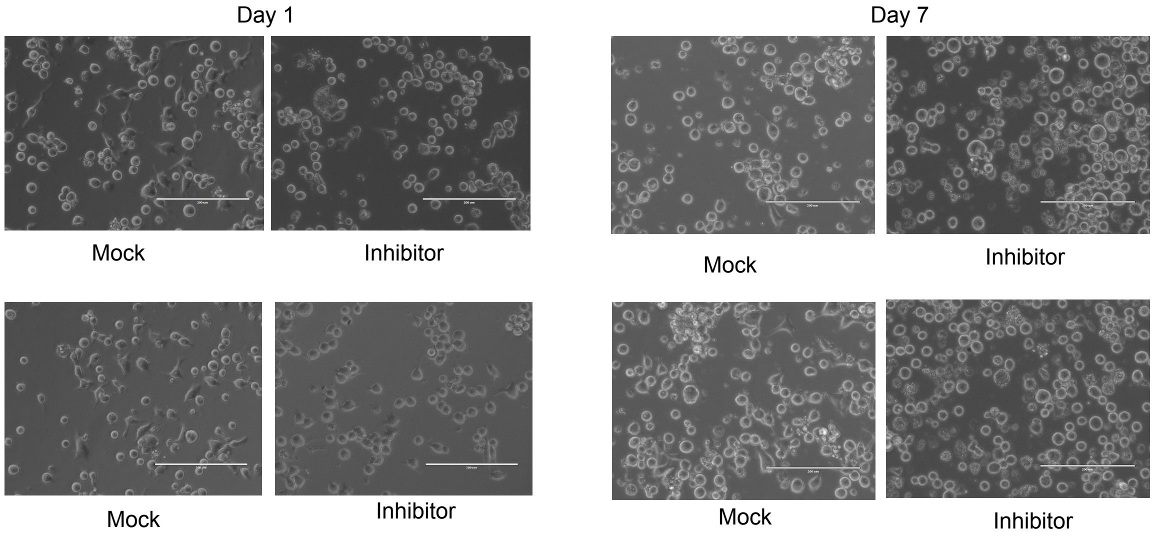

Supplementary Figure B

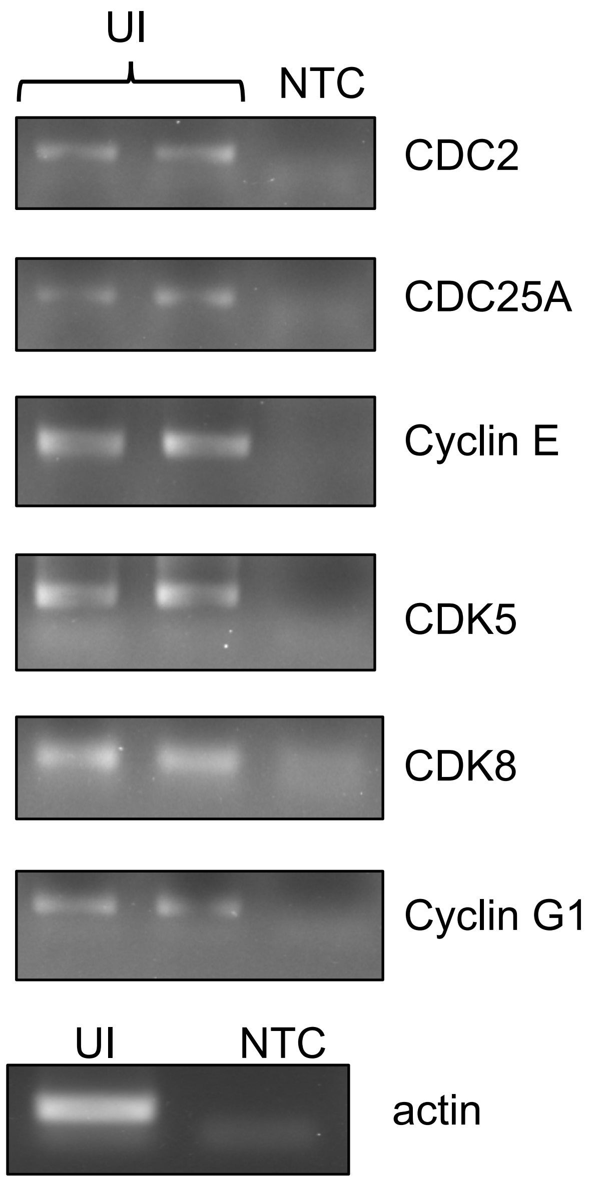

Supplementary Figure C

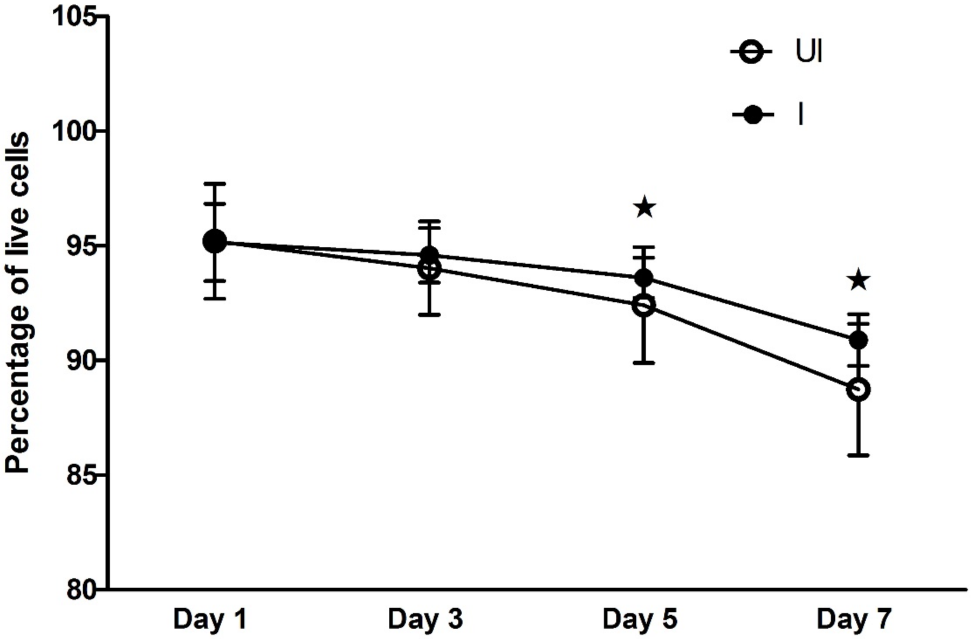

Supplementary Figure D

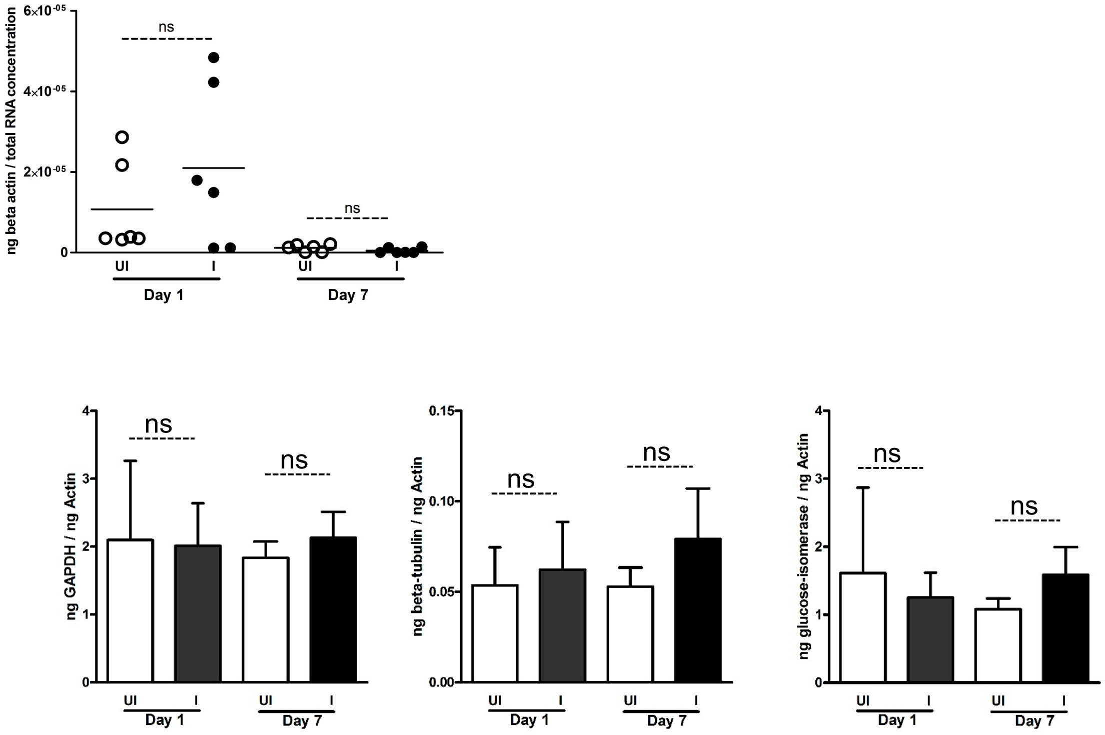

Supplementary Figure E

Supplementary Table A: Primer sequences used in QRT-PCR analysis

Primer sequence (5'-3')	gene, purpose
AAACTACAGGTCAAGTGGTAGCC<br>AATCCTGCATAAGCACATCC	CDC2, qPCR
GTGAAGGCGCTATTTGGCG<br>GGTCCATAGTGACGGTCAGGT	CDC25A, qPCR
GTTATAAGGGAGACGGGGAG<br>TGCTCTGCTTCTTACCGCTC	Cyclin E, qPCR
AGAGGAGCCGACCCGTTGC<br>TCCATTCCCCACCTTCTTCCC	CDK5, qPCR
GGGATCTCTATGTCGGCATGTAG<br>AAATGACGTTTGGATGCTTAAGC	CDK8, qPCR
TTCCAAGATA AATGGCAGAG<br>GCAGTACGCCCAGAAACA	Cyclin G1, qPCR
AGCCTCGCCTTTGCCGA<br>CTGGTGCCTGGGGCG	Actin, qPCR
CCAGCGTTTAGCAAGATAAGAG<br>GCCCAGTAACAACATCATAAGC	*Anaplasma p44 gene,* qPCR
ACAGTCCATGCCATCACTGCC<br>GCCTGCTTCACCACCTTCTTG	GAPDH, qPCR
GCATTCGATCTAGCAAATTAGGAGCTC<br>CTCAATCAGCTGGTGGATAGACAGAACC	Beta-1-tubulin, qPCR
AGGCTGCTGCCACATAAGGT<br>CCAAGGCTCCAAGCATGAAT	Glucose-6-isomerase, qPCR
